# Development of *neffy*, an Epinephrine Nasal Spray, for Severe Allergic Reactions

**DOI:** 10.3390/pharmaceutics16060811

**Published:** 2024-06-14

**Authors:** Anne K. Ellis, Thomas B. Casale, Michael Kaliner, John Oppenheimer, Jonathan M. Spergel, David M. Fleischer, David Bernstein, Carlos A. Camargo, Richard Lowenthal, Sarina Tanimoto

**Affiliations:** 1 Division of Allergy and Immunology, Department of Medicine, Queen’s University, Kingston, ON K7L 3N6, Canada; 2 Morsani College of Medicine, University of South Florida, Tampa, FL 33602, USA; 3 Institute for Asthma and Allergy, Chevy Chase, MD 20815, USA; 4 Department of Internal Medicine, University of Medicine and Dentistry of New Jersey-Rutgers New Jersey Medical School, Newark, NJ 07103, USA; 5 Division of Allergy and Immunology, Children’s Hospital of Philadelphia, Department of Pediatrics, Perelman School of Medicine, University of Pennsylvania, Philadelphia, PA 19104, USA; 6 Section of Allergy and Immunology, Department of Pediatrics, Children’s Hospital Colorado, School of Medicine, University of Colorado, Aurora, CO 80045, USA; 7 Bernstein Clinical Research Center, Division of Immunology, Allergy and Rheumatology, College of Medicine, University of Cincinnati, Cincinnati, OH 45236, USA; 8 Department of Emergency Medicine, Massachusetts General Hospital, Harvard Medical School, Boston, MA 02114, USA; 9 ARS Pharmaceuticals, Inc., San Diego, CA 92130, USA

**Keywords:** epinephrine, anaphylaxis, severe allergy, food allergy, nasal spray, intranasal epinephrine, out of hospital use, pharmacokinetics, pharmacodynamics

## Abstract

Epinephrine autoinjectors (EAIs) are used for the treatment of severe allergic reactions in a community setting; however, their utility is limited by low prescription fulfillment rates, failure to carry, and failure to use due to fear of needles. Given that delayed administration of epinephrine is associated with increased morbidity/mortality, there has been a growing interest in developing needle-free, easy-to-use delivery devices. ***neffy*** (epinephrine nasal spray) consists of three Food and Drug Administration (FDA)-approved components: epinephrine, Intravail A3 (absorption enhancer), and a Unit Dose Spray (UDS). ***neffy***’s development pathway was established in conjunction with the FDA and the European Medicines Agency and included multiple clinical trials to evaluate pharmacokinetic and pharmacodynamic responses under a variety of conditions, such as self-administration and allergic and infectious rhinitis, as well as an animal anaphylaxis model of severe hypotension, where ***neffy*** demonstrated a pharmacokinetic profile that is within the range of approved injection products and a pharmacodynamic response that is as good or better than injections. The increased pulse rate (PR) and blood pressure (BP) observed even one minute following the administration of ***neffy*** confirm the activation of α and β adrenergic receptors, which are the key components of epinephrine’s mechanism of action. The results suggest that ***neffy*** will provide a safe and effective needle-free option for the treatment of severe allergic reactions, including anaphylaxis.

## 1. Introduction

There is a growing interest in using intranasal (IN) administration to administer a variety of therapeutics, particularly for out-of-hospital use. In addition to being much less invasive, IN administration offers multiple advantages over other routes of administration, including ease of use, rapid absorption, and avoidance of pain typically associated with intravenous (IV) or intramuscular (IM) injection. Needle-free delivery options are particularly beneficial for children, who are more likely to be “needle-phobic”. A range of medications, including midazolam, diazepam, fentanyl, naloxone, ketamine, and dexmedetomidine, among others, are routinely administered intranasally for a variety of indications [1,2,3,4,5,6,7,8,9].

The vast majority of severe Type I allergic reactions occur in out-of-hospital settings, and the immediate administration of epinephrine is the only universally recommended first-line treatment [10,11,12]. Antihistamine and corticosteroid agents are considered second-line treatment for anaphylaxis, given their slow onset of action and inability to stabilize or prevent mast cell degranulation or to target additional mediators of anaphylaxis, which is the essential physiologic effects of epinephrine [10]. Although epinephrine autoinjectors (EAIs) are the most commonly prescribed products for community-based epinephrine therapy, fewer than half of patients at risk for severe allergic reactions (including anaphylaxis) actually carry the products with them on a regular basis, and those that do often delay use during a severe Type I allergic reaction [13,14]. Failed or delayed treatment is associated with significant increases in the risks of biphasic reactions, hospitalization, and death [14,15,16,17,18,19]. The primary reason cited for failed/delayed treatment is needle-phobia [13,14,20].

Low utilization rates, particularly in light of the serious adverse outcomes associated with failed/delayed treatment, represent a significant unmet medical need for patients at risk for severe allergic reactions, including anaphylaxis. To address these needs, ARS Pharmaceuticals, Inc. (ARS) is developing an epinephrine nasal spray, ***neffy***, which is currently under review by the United States (US) Food and Drug Administration (FDA) and the European Medicines Agency (EMA). This report provides an introduction to ***neffy***, including its development strategy and a review of published clinical data.

## 2. *neffy* Product Information

**Figure 1:**
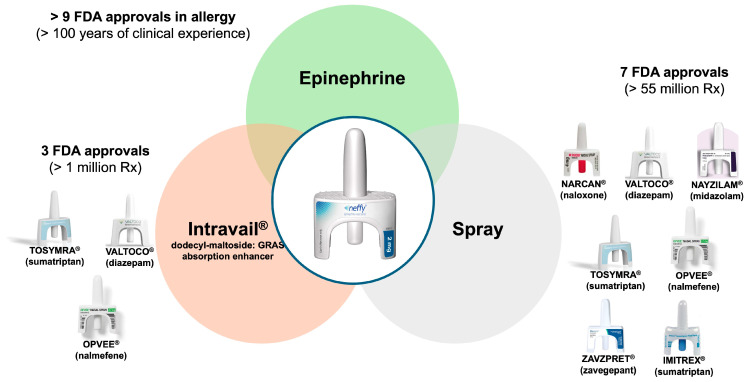
***neffy***’s development rests upon the proven triad of epinephrine, Intravail, and a Unit Dose Spray to ensure a safe and effective product.

***neffy*** (epinephrine nasal spray) is a combination of three FDA-approved components, including (1) epinephrine, the active ingredient; (2) Intravail (dodecylmaltoside [DDM]), a proprietary absorption-enhancing agent called to improve the bioavailability of IN-administered drugs; and (3) a Unit Dose Spray (UDS) designed to produce a spray pattern and droplet size that maximizes delivery to the turbinate (Figure 1).

### 2.1. Epinephrine

Epinephrine has been used for allergic reactions for more than 100 years and is the only universally recommended first-line therapy for Type I allergic reactions. The use of epinephrine for the treatment of anaphylaxis was first reported in the 1960s. It is agreed internationally that epinephrine is the most effective treatment for anaphylaxis based on clinical guidelines based on vast experience, case reports, and limited clinical trials [21,22,23,24,25,26,27,28].

Epinephrine’s mechanism of action (MOA) for the treatment of Type I allergic reactions (including anaphylaxis) is generally well understood, where the reverse of the pathological response caused by exposure to an antigen and a stabilized mast cell to stop allergic reactions from proceeding are based on the direct systemic agonism of α- and β-adrenergic receptors [29].

Epinephrine is a nonselective agonist at the α- and β-adrenergic receptors, which are all G-protein-coupled receptors. Epinephrine prevents further degranulation and release of allergic mediators within minutes by counteracting nearly every end-organ action of immune mediators of anaphylaxis directly and stabilizing mast cells [30]. The main therapeutic effect of epinephrine arises from its direct agonism of β_2_-adrenergic receptors, resulting in the activation of adenylyl cyclase and increased intracellular cyclic AMP production [31]. While anaphylaxis leads to the loss of intravascular fluid volume and hypotension, α-adrenergic receptors reduce vasodilation and increase vascular permeability. β-adrenergic receptors relax bronchial smooth muscle and help alleviate bronchospasm, wheezing, and dyspnea that may occur during anaphylaxis. Heart rate and contractility increase via β-adrenergic receptors to maintain the blood pressure (BP). Having the ability to produce relaxation effects on the smooth muscle of the stomach, intestine, uterus, and urinary bladder, epinephrine improves symptoms such as pruritus, urticaria, and angioedema and may relieve gastrointestinal and genitourinary symptoms associated with anaphylaxis [32].

Anaphylaxis most often occurs in response to food, insect stings, and drugs but can also be exercise-induced or idiopathic. Because anaphylaxis can occur outside the home, patients should be counseled on allergen avoidance and the importance of having epinephrine available [12]. Effective symptom resolution, in part, depends on the immediate administration of epinephrine by a patient or caregiver [29]. At the same time, the risk of overdose and thus severe cardiac adverse effects, while possible with any injection route of administration [33], is lower with IM administration than with IV administration.

The FDA has approved several IM and subcutaneous epinephrine injection and EAI products, including EpiPen^®^ (Mylan Specialty L.P., Morgantown, WV, USA), Twinject^®^ (Amedra Pharmaceuticals LLC, Horsham, PA, USA), Adrenaclick^®^ (Lineage Therapeutics Inc., Horsham, PA, USA), Auvi-Q^®^ (Kaleo, Inc., Richmond, VA, USA), and Symjepi^TM^ (Adamis Pharmaceuticals Corporation, San Diego, CA, USA), as well as generic EAIs [34]. With the exception of one pharmacokinetic study conducted for Auvi-Q [35], there were no clinical trials or pharmacokinetic studies conducted to support the approval of these products. Instead, their approval was based on the assumption that there were no significant differences between these injection products and the reference listed drug (IM injection with needle and syringe). However, more recent studies have established notable pharmacokinetic differences among the different autoinjector products and manual IM injection with needle and syringe [36,37,38]. 

It is important to note, however, that these pharmacokinetic differences do not appear to translate into differences in clinical efficacy. All approved injection products are used interchangeably, with the same guidance (dose immediately upon the development of clinical symptoms, with a second dose 5 to 15 min later if symptoms do not improve).

### 2.2. Intravail

Intravail, also referred to as DDM, is designated as Generally Recognized As Safe (GRAS) for food applications and is used to enhance the absorption of drugs such as epinephrine. It has been used in several FDA-approved drugs, including Tosymra^®^ (Dr. Reddy’s Laboratories Limited, Princeton, NJ, USA), Valtoco^®^ (Neurelis, Inc., San Diego, CA, USA), and Opvee^®^ (Opiant Pharmaceuticals, Santa Monica, CA, USA) [39]. Intravail is an alkylsaccharide that alters mucosal viscosity and membrane fluidity to loosen cell–cell junctions. It induces rapid and reversible decreases in transepithelial/transendothelial electrical resistance values, resulting in changes to the tight junctions to facilitate absorption [40,41]. Alkylsaccharide absorption enhancers are soluble in both water and oil and do not cause irritation or damage to the mucosal membrane [6].

The use of an absorption enhancer like Intravail allows the dose of epinephrine to remain as low as possible while maintaining efficacy and allows for a lower dose relative to other IN formulations without a comparable absorption enhancer [42,43,44,45]. There may be some concerns that epinephrine’s vasoconstriction effect may negatively affect its absorption, but such an effect has not been observed with ***neffy*** with Intravail. The mechanism may be discussed further in the future, but at least epinephrin is not just a vasoconstrictor but also a vasodilator. Although epinephrine is considered the only safe and effective first-line treatment of anaphylaxis [46], there have been reports of safety issues due to overdosing, both following manual IM administration and EAI administration [29,47,48]. For IN products, it has been reported that epinephrine absorption during an allergic reaction may be increased due to changes in vascular permeability [49,50]. A high dose of epinephrine in the presence of increased permeability in the nasal mucosa, due to an allergic reaction or population variability, could potentially result in excessive absorption and increase the risk of overdose [51]. The inclusion of Intravail in the ***neffy*** formulation allows for the optimization of efficacy while minimizing the risk of overdose by capping the dose. 

Use of the lowest possible epinephrine dose, or minimum effective dose, also reduces the risk of dose-limiting toxicities. These doses minimize the risk of other adverse reactions, including gastrointestinal side effects such as abdominal pain, nausea, and vomiting, which could complicate the diagnosis and treatment of anaphylaxis. In particular, dose-limiting gastrointestinal side effects have been observed with large doses of inhaled epinephrine [52]. Additionally, a review of patients with epinephrine toxicity defined a maximum tolerated subcutaneous dose of 8 mg [53].

The ability to use a dose of epinephrine that was as low as possible, while still achieving injection-like exposure, was a key part of ***neffy****’s* development strategy and may be an important consideration for the evaluation of emerging epinephrine therapies [51].

### 2.3. Unit Dose Spray

***neffy*** is delivered via a UDS that has been used for more than 20 years for the administration of numerous other IN medications, including Narcan^®^ (over the counter) (Adapt Pharma, Inc., Radnor, PA, USA), Valtoco^®^ (Neurelis, Inc., San Diego, CA, USA), Nayzilam^®^ (UCB, Inc., Smyrna, GA, USA), Tosymra^®^ (Dr. Reddy’s Laboratories Limited, Princeton, NJ, USA), Imitrex^®^ (GlaxoSmithKline, Research Triangle Park, Durham, NC, USA), Zavzpret^®^ (Pfizer Inc., New York, NY, USA), and Opvee^®^ (Opiant Pharmaceuticals Santa Monica, CA, USA). The device is easy to use and highly reliable, with a failure rate of less than 1 in 100,000 uses across several million prescriptions (real-world failure rate of 0.3 per 1,000,000 devices for Narcan) [54,55]. The UDS is designed to deliver more than 80% of the drug in droplets measuring between 20 and 120 µm, almost all of which are exclusively captured on the nasal turbinates [56]. However, there could be issues in product use, such as spraying before positioning the spray in the nose; therefore, instructions to use it need to be referred to.

## 3. *neffy* Development Strategy

Ethical and practical limitations preclude the conduct of randomized controlled trials to assess the efficacy of epinephrine products for the treatment of severe Type I allergic reactions (including anaphylaxis), and to date, no such trials have been conducted [57,58]. There are several reasons for the lack of such studies. First, the unpredictable clinical course. When an allergic reaction occurs, it is difficult to impossible to predict the progression, severity, and likelihood of fatality [59,60,61], which is the case even in the same patient from one allergic reaction to another [62,63]. Such unpredictability of the clinical course could put patients at risk of life-threatening, potentially fatal conditions [30,47,64,65,66]. Second, given the high degree of variability in severe Type I allergic reactions (e.g., type of allergen and clinical course) and the relative infrequency of anaphylaxis, a large study population would be required to achieve sufficient statistical power [64,67]. Lastly, experience with epinephrine use over 100 years has demonstrated its safety and efficacy in even severe anaphylaxis using any route of administration. 

In addition to the lack of efficacy trials, the pharmacokinetics of acute epinephrine administration have not been well characterized, with previous work being based on IV infusion. With the exception of one pharmacokinetic study conducted with Auvi-Q and EpiPen [35], the current EAIs were approved without conducting any clinical trials. Recent pharmacokinetic studies were conducted under the directive of the EMA in 2015 and by ARS for the development of ***neffy*** and have demonstrated that there are significant differences in pharmacokinetic profiles among approved injection products [36,37,38].

Therefore, over the course of 8 years, ARS worked closely with the FDA and the EMA to create a development pathway to evaluate ***neffy****’s* safety and efficacy. This pathway has included multiple clinical trials to evaluate the pharmacokinetic and pharmacodynamic response of ***neffy*** in controlled settings under a variety of potential real-life conditions, including self-administration, allergic rhinitis, and infectious rhinitis, as well as severe hypotension in an animal model.

## 4. Review of *neffy* Data

This review includes published data from several clinical trials conducted as part of ***neffy****’s* development program. All study protocols were approved by the relevant Institutional Review Boards or Ethics Committees and all participants gave written informed consent before study participation. The studies were conducted according to the International Conference on Harmonization Guidelines for Good Clinical Practice. 

Blood samples for pharmacokinetic analysis were collected before dosing and at 2, 4, 6, 8, 10, 12.5, 15, 20, 30, 45, 60, 90, 120, 150, 180, and 240 (360 and 480 min depending on the study) minutes after dosing. Plasma epinephrine concentrations were determined using a validated liquid chromatography–mass spectrometry method. Pharmacodynamic parameters, including systolic blood pressure (SBP), diastolic blood pressure (DBP), and pulse rate (PR), were measured using an automated BP measuring device. BP and PR were measured at baseline; before dosing; and at 1 (depending on the study), 5, 10, 15, 20, 25, 30, 45, 60, 90, and 120 min after dosing.

### 4.1. neffy 1 mg Studies in Humans

***neffy****’s* initial development began with a proposed 1 mg dose. An integrated analysis was conducted using data from four randomized cross-over Phase 1 trials (n = 175) comparing the pharmacokinetics and pharmacodynamics of manual IM epinephrine 0.3 mg with needle and syringe (epinephrine 0.3 mg IM), epinephrine 0.3 mg autoinjectors (Symjepi 0.3 mg and EpiPen 0.3 mg), and ***neffy*** 1 mg. Two studies enrolled healthy individuals aged 19 to 55 years, and the other two studies enrolled healthy volunteers with a history of type I allergies (allergic rhinitis, food allergy, venom allergy), aged 19 to 55 years [36]. 

In this integrated analysis, ***neffy*** 1.0 mg demonstrated a pharmacokinetic profile that was comparable to what was observed following manual IM injection but less than what was observed following EAIs. ***neffy****’s* pharmacodynamic profile was comparable to what was observed following EAIs.

Pharmacokinetics

**Figure 2:**
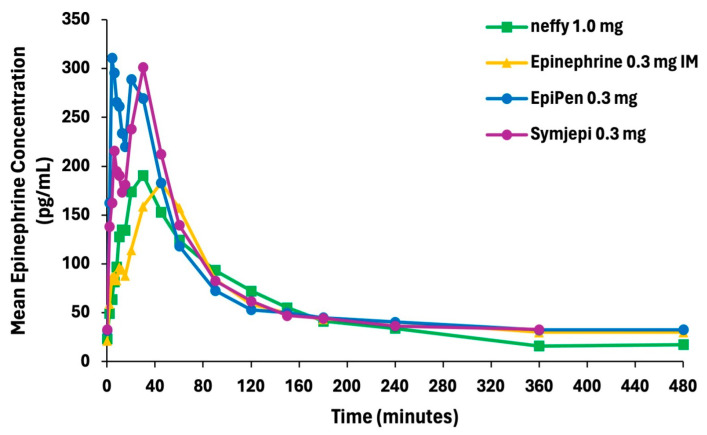
Mean plasma epinephrine concentration vs. time after ***neffy***, EpiPen, Symjepi, and epinephrine 0.3 mg intramuscular (IM).

The epinephrine concentration vs. time curve showed the highest mean epinephrine concentration after administration through EpiPen, followed by Symjepi, ***neffy***, and epinephrine 0.3 mg IM (Figure 2).

Pharmacodynamics

**Figure 3:**
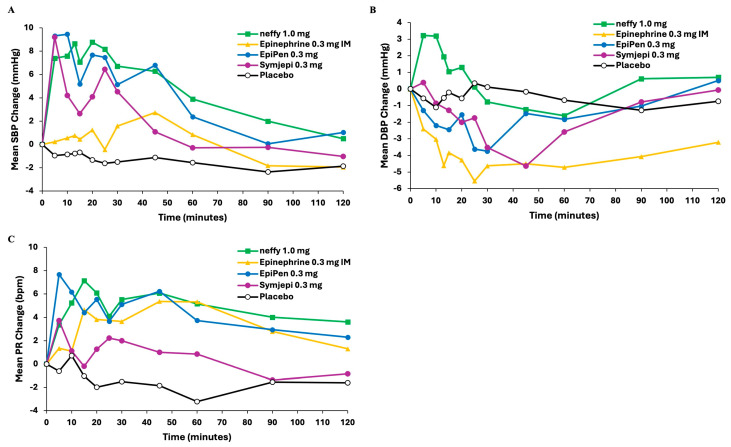
Pharmacodynamic measurements vs. time after ***neffy***, EpiPen, Symjepi, epinephrine 0.3 mg IM, and placebo: (**A**) Mean change from baseline in systolic blood pressure (SBP). (**B**) Mean change from baseline in diastolic blood pressure (DBP). (**C**) Mean change from baseline in pulse rate (PR).

EpiPen, Symjepi, and ***neffy*** resulted in comparable increases in mean SBP vs. time, whereas the change with epinephrine 0.3 mg IM was less pronounced (Figure 3). For DBP, ***neffy*** was the only product that resulted in an increase in mean value over time. In all injection products, there was a decrease in DBP, with the magnitude of decrease after epinephrine injection being greater than that observed after placebo (Figure 3). The peak mean PR vs. time was the greatest for EpiPen, followed by ***neffy***, epinephrine 0.3 mg IM, and Symjepi (Figure 3). 

In this analysis, ***neffy*** led to a modestly more robust increase for SBP, despite having lower or comparable peak concentration relative to injection products. This greater effect on SBP may be attributed to the difference in activating the β_2_ receptors that are abundant in skeletal muscles, allowing them to be preferentially activated by direct IM injection of epinephrine (through either manual injection or autoinjector administration). The β_2_ adrenergic receptors have the highest affinity and are activated at relatively low epinephrine concentrations. They promote vasodilation in the skeletal muscle, causing a decrease in peripheral vascular resistance and increased blood flow to skeletal muscle in the thigh, ultimately resulting in a decrease in DBP, which may drive an attenuation of the increase in SBP.

### 4.2. neffy 2 mg Studies in Human

#### 4.2.1. Studies in Healthy Subjects

##### Crossover Study Comparing ***neffy*** 2.0 mg vs. EpiPen and Manual IM Injection—Dosing Once and Twice

This was a Phase 1 crossover study with healthy subjects conducted to evaluate the pharmacokinetics and pharmacodynamics of ***neffy*** 2.0 mg compared with EpiPen 0.3 mg and manual IM epinephrine 0.3 mg with needle and syringe (epinephrine 0.3 mg IM). The objective of this study was to demonstrate that the pharmacokinetic and pharmacodynamic profiles of ***neffy*** were within the range of approved epinephrine injection products. A total of 59 subjects aged 21 to 54 years old received a single dose of ***neffy***, EpiPen, and epinephrine 0.3 mg IM, and a repeat dose of ***neffy*** and EpiPen were analyzed [68]. 

This study demonstrated that ***neffy*** 2 mg has a pharmacokinetic profile within the range of currently approved epinephrine injection products and a pharmacodynamic profile that was comparable to or better than injection products.

##### Pharmacokinetics

**Figure 4:**
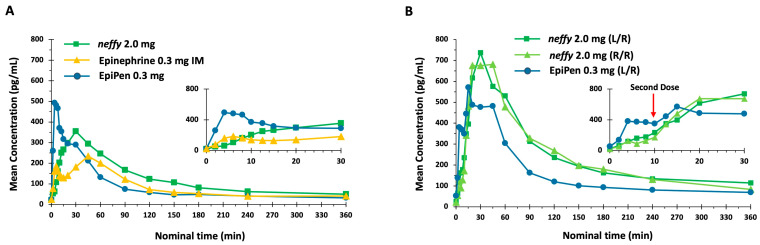
Mean epinephrine concentration time. Study conducted on healthy volunteers. N = 42 for ***neffy*** 2.0 mg, epinephrine 0.3 mg IM, and EpiPen 0.3 mg. N = 39 for ***neffy*** 2.0 mg (L/R) and ***neffy*** 2.0 mg (R/R). N = 42 for EpiPen 0.3 mg (L/R) [68]. (**A**) Single dose. (**B**) Repeat dose with second dose administered at 10 min.

Mean epinephrine concentrations were highest following a single EpiPen dose, which persisted until approximately 20 min after dosing (Figure 4). From 30 to 360 min after dosing, greater mean epinephrine concentrations were observed following ***neffy*** relative to both EpiPen and epinephrine IM. Following repeated doses, greater mean epinephrine concentrations were observed with both ***neffy*** treatments (R/R and L/R) compared with EpiPen.

##### Pharmacodynamics

**Figure 5:**
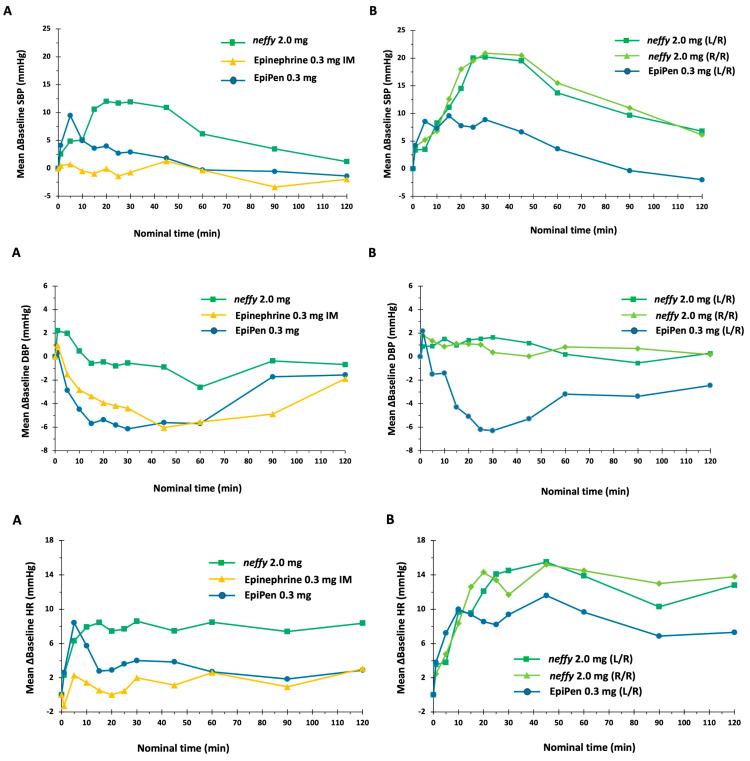
Pharmacodynamic measurements vs. time after ***neffy***, EpiPen, and epinephrine 0.3 mg IM: (**A**) Single doses. (**B**) Repeat doses.

***neffy***’s pharmacodynamic response on SBP was observed starting at one minute after administration and persisted for 120 min (Figure 5). EpiPen was associated with a less pronounced and more abrupt increase in SBP relative to ***neffy***; a nominal change in SBP was observed following epinephrine IM. For all treatments, SBP returned to baseline approximately 120 min after dosing. For DBP, all treatments resulted in an immediate increase from baseline, followed by a decrease (Figure 5). The decrease was more pronounced following EpiPen and Epinephrine IM compared with ***neffy***, which was consistent with what was observed in the integrated analysis in Section 4.1. All treatments resulted in an increase from baseline PR (Figure 5). The initial increase was followed by a decrease for both epinephrine IM and EpiPen, whereas the elevation persisted throughout 120 min following ***neffy.***

##### Crossover Study Comparing ***neffy*** 2.0 mg (Self-Administration) vs. Manual IM Injection (via Healthcare Provider)

This was a Phase 1 crossover study in adults with Type I allergies conducted to evaluate the pharmacokinetics and pharmacodynamics of self-administered ***neffy*** 2 mg compared with health care provider (HCP)-administered manual IM epinephrine 0.3 mg with needle and syringe [69]. Given that ***neffy*** is intended for use both in and outside of hospital settings, it was necessary to illustrate ***neffy****’s* pharmacokinetics and pharmacodynamics following self-administration. A total of 45 patients aged 23 to 53 years old with a history of type I allergy were enrolled [69].

Following self-administration, ***neffy*** 2.0 mg resulted in pharmacokinetic and pharmacodynamic profiles that were comparable to, or better than, HCP-administered epinephrine 0.3 mg IM, including a more pronounced increase in SBP following ***neffy***. These data were consistent with other studies presented in this review and demonstrated that, following self-administration, ***neffy***’s pharmacokinetic and pharmacodynamic profiles are within the range of injection products.

##### Pharmacokinetics

**Figure 6:**
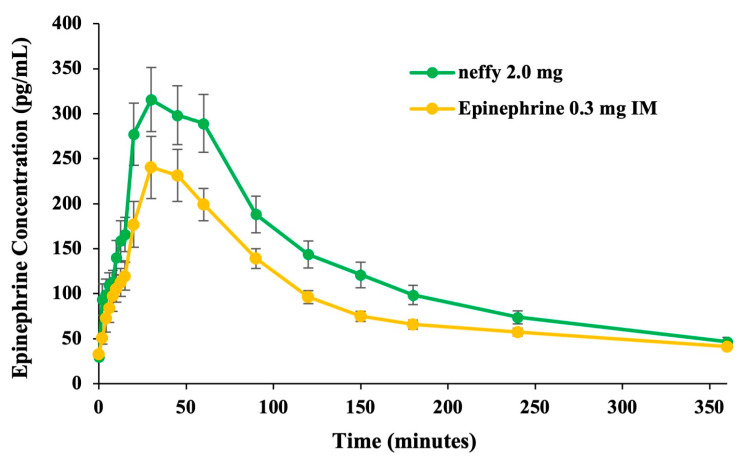
Mean epinephrine concentration time. Study conducted on patients with a history of Type 1 allergy. N = 43 for ***neffy*** 2.0 mg via self-administration and epinephrine 0.3 mg IM via HCP [69]. Error bars represent standard error.

Overall, when self-administered, ***neffy*** resulted in higher epinephrine exposures relative to HCP-administered epinephrine 0.3 mg IM (Figure 6).

##### Pharmacodynamics

**Figure 7:**
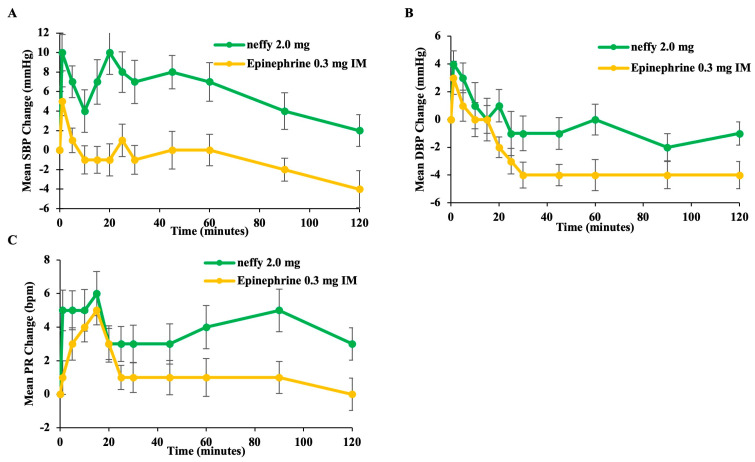
Pharmacodynamic measurements vs. time after ***neffy*** and epinephrine 0.3 mg IM: (**A**) Mean change from baseline in SBP. (**B**) Mean change from baseline in DBP. (**C**) Mean change from baseline in PR. Error bars represent standard error.

Compared with HCP-administered IM 0.3 mg, self-administered ***neffy*** resulted in a greater mean increase from baseline SBP, DBP, and PR, and the pharmacodynamic response was observed as soon as one minute after administration (Figure 7).

#### 4.2.2. Studies on Patients with Different Conditions

##### Crossover Study Comparing ***neffy*** 2.0 mg under Normal Nasal Conditions and during Infectious Rhinitis

This was a Phase 1 study in subjects during and after upper respiratory tract infections (URTIs). Given the high prevalence of URTIs, this study was conducted to characterize ***neffy’s*** pharmacokinetics and pharmacodynamics during active URTI conditions. The pharmacokinetics and pharmacodynamics of ***neffy*** 2 mg were assessed during an active URTI and again upon recovery (normal nasal conditions) [70]. Subjects were enrolled during symptoms of URTIs with positive nasal congestion and edema (Total Nasal Symptom Score [TNSS] of ≥5 out of 12 and a congestion score of ≥2 out of 3). A single dose of ***neffy*** 2.0 mg was administered during the URTI, followed by pharmacokinetic and pharmacodynamic assessments. Subjects returned after recovery from URTI to receive a second dose under normal nasal conditions (TNSS score of ≤2 out of 12 and a congestion score of ≤1 out of 3), followed by repeated pharmacokinetic and pharmacodynamic assessments. Subjects who used oral and/or nasal decongestants within 24 h before dosing were not enrolled. A total of 21 patients aged 19 to 55 years old were enrolled.

##### Pharmacokinetics

**Figure 8:**
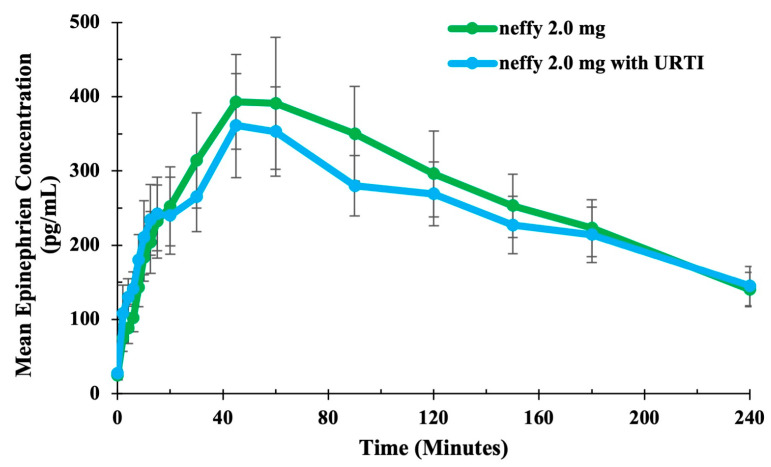
Mean epinephrine concentration time after ***neffy*** by nasal condition. Study conducted on patients who developed URTI. N = 21 for ***neffy*** 2.0 mg with URTI. N = 16 for ***neffy*** 2.0 mg under normal conditions [70]. Error bars represent standard error.

The study demonstrated that URTIs had minimal impact on the absorption of or maximum exposure to epinephrine or following administration of ***neffy*** 2.0 mg (Figure 8). 

##### Pharmacodynamics

**Figure 9:**
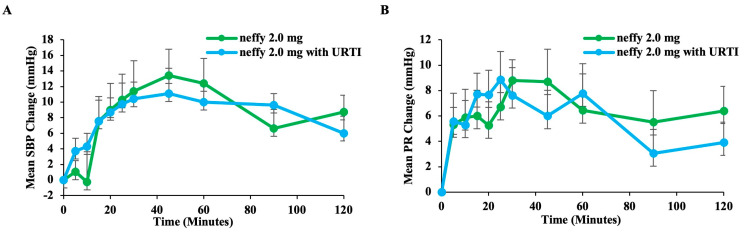
Pharmacodynamic response to ***neffy*** by nasal condition: (**A**) Mean change from baseline in SBP. (**B**) Mean change from baseline in PR. Error bars represent standard error.

The mean change from baseline SPB and PR values was similar between URTIs and normal conditions, and a pharmacodynamic response was observed as soon as one minute after administration (Figure 9).

##### Effect of Allergic Rhinitis

Based on feedback from the FDA, ARS conducted an additional study to assess the impact of Nasal Allergy Challenge (NAC)-induced allergic rhinitis on the comparative bioavailability of ***neffy*** 2 mg versus manual IM epinephrine 0.3 mg IM with needle and syringe (epinephrine 0.3 mg IM) under normal nasal conditions. 

This was a Phase 1 study in subjects with a history of allergic rhinitis. The NAC was conducted at screening, with eligibility limited to subjects who had a TNSS of ≥5 out of 12 and a congestion score of ≥2 out of 3. A total of 36 subjects aged from 20 to 52 years old were enrolled. 

**Figure 10:**
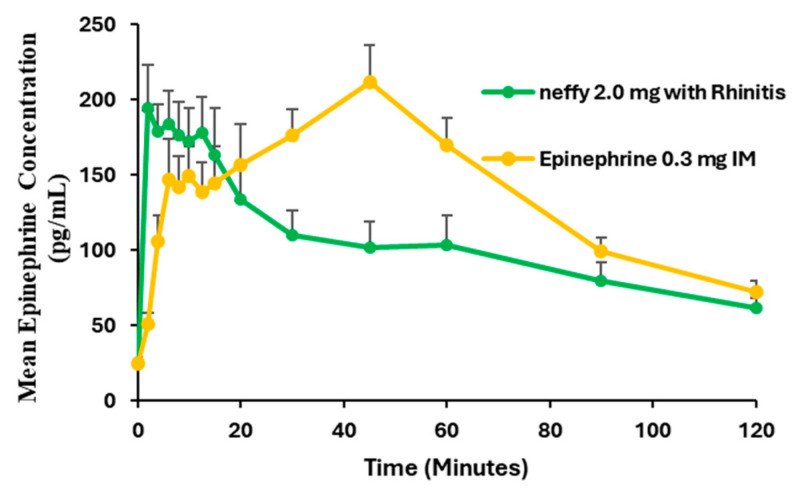
Mean epinephrine concentration time. Study conducted on patients with a history of seasonal allergic rhinitis. N = 34 for neffy 2.0 mg with rhinitis. N = 35 for epinephrine 0.3 mg IM. Error bars represent standard error.

Administration of a single dose of ***neffy*** following NAC-induced allergic rhinitis resulted in an increase in the rate of epinephrine absorption. It is assumed that this increased absorption is due to the increased permeability in the nasal mucosa, as this phenomenon was observed in the anaphylaxis dog model and has been reported in the literature [49,50]. At the same time, ***neffy*** 2 mg with rhinitis also resulted in more rapid clearance compared with normal nasal conditions (Figure 10). 

While these data demonstrated that there appears to be sufficient epinephrine exposure with ***neffy*** (i.e., greater than 0.3 mg IM epinephrine injection) at the critical early time points, including the first 5 to 15 min when reversal of the initial symptoms of allergic reactions is typically observed with a single dose, an additional clinical study was developed in conjunction with the FDA to assess ***neffy’s*** pharmacokinetics following dosing twice during these same NAC-induced allergic rhinitis conditions. The results of this study were being reviewed by the FDA at the time this report was produced.

#### 4.2.3. Anaphylaxis Dog Study

The EMA requested data on ***neffy***’s absorption during severe hypotension. Because it is not possible to induce severe hypotension in human subjects, a Good Laboratory Practice (GLP) study was conducted in anesthetized beagle dogs. The objective of this GLP study was to evaluate ***neffy***’s pharmacokinetics in the dogs under both normal and Tween 80-induced anaphylaxis conditions [71]. A total of 14 dogs (10 males and 4 females) were dosed with ***neffy*** 1.0 mg under normal conditions, followed by ***neffy*** 1.0 mg under anaphylaxis conditions with severe hypotension [49]. The mean (±SD) baseline SBP/DBP was 113 (±47)/62 (±27) mm Hg before anesthesia induction, with a decrease to 94 ± 16/55 ± 13 mm Hg following general anesthesia. For the anaphylaxis session, the mean (±SD) SBP/DBP was 137 (±50.4)/78 (±30) mm Hg before anesthesia induction (and Tween 80 administration), with a decrease to 61 (±10)/39 (±7) mm Hg following anesthesia induction and Tween 80 administration. The more pronounced decrease seen during anaphylaxis represents the combined effect of anesthesia and anaphylaxis.

The results of this study demonstrated that the absorption of epinephrine was not suppressed even during anaphylaxis with severe hypotension and was, in fact, increased. This may be because vasoactive mediators such as histamine released during anaphylaxis increase vascular permeability [72,73]. Increased epinephrine absorption has also been reported under histamine-induced nasal congestion in dogs [50]. These data suggest that IN absorption of epinephrine may be enhanced during the increased permeability associated with a more severe anaphylaxis event.

**Figure 11:**
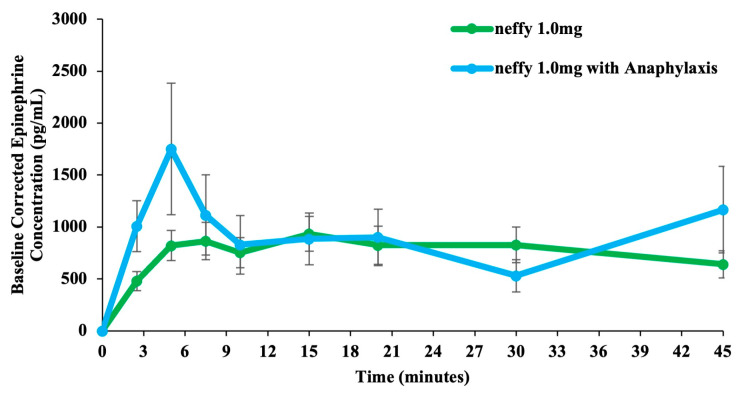
Mean epinephrine concentration-time. Study conducted on dogs. N = 14 neffy 1.0 mg under normal conditions. N = 12 for neffy 2.0 mg with anaphylaxis [49]. Error bars represent standard error. Note: baseline at −3 min is shifted to 0 in the figure.

Anaphylaxis induction resulted in a marked increase in epinephrine concentrations, which were pronounced at early time points (Figure 11).

## 5. Conclusions

The low utilization rates of current EAIs represent a significant unmet medical need among patients at risk for severe allergic reactions, including anaphylaxis. Prompt treatment with epinephrine at the first symptom/sign is critical to stop disease progression. This is even more important when the clinical course is unpredictable and can initially present as mild. Failed or delayed treatment is associated with an increased risk of severe anaphylaxis, biphasic reactions, hospitalization, and death. The limitations of the currently approved EAIs are generally attributable to needle-phobia, the bulkiness of carrying an EAI, and patients’ own safety concerns, which speak to the need for additional treatment options. 

Nasal administration of epinephrine may be an attractive option, providing patients and caregivers with a needle-free, pain-free delivery option that results in rapid absorption and resolution of symptoms. ***neffy***’s development is the result of more than 8 years of close collaboration with the FDA and rests upon the proven triad of epinephrine, Intravail, and a UDS to ensure a safe and effective product.

Across a range of studies, ***neffy*** has demonstrated a pharmacokinetic profile that is within the range of currently approved injection products, as well as pharmacodynamic responses that are as good or better than injection, including a response observed as early as one minute after treatment, which confirms activation of α- and β-adrenergic receptors that underlie epinephrine’s MOA for the treatment of allergic reactions. Importantly, these findings were reproduced under a variety of nasal conditions, including moderate to severe congestion and/or rhinorrhea due to allergic rhinitis and infectious rhinitis. The results of the GLP dog study demonstrate that epinephrine administered via ***neffy*** is effectively absorbed despite severe hypotension caused by anaphylaxis. 

***neffy***’s safety and efficacy are anticipated to be comparable to current injection products while providing patients and caregivers with a treatment option that results in immediate receptor activation and removes the most significant barriers to use. By reducing a patient’s hesitation to treat themselves with epinephrine, ***neffy*** should increase earlier use of epinephrine and thereby reduce the risk of progression to severe anaphylaxis.

## Data Availability

Not applicable.
